# Dr. Domeier, on the Appearance of the Tongue

**Published:** 1800-11

**Authors:** W. Domeier

**Affiliations:** London


### Dr. DOMEIER, *on the Appearance of the Tongue.*

" *Is a furred tongue a sign of a foul stomach?*"

IN examining the hiſtory of medicine, it will not eſcape our attention, that one of the ſtrongeſt reaſons which retarded its progreſs, was our ſwearing in verba magiſtri. Whatever part, eſpecially in the practice of medicine, we conſider, we find this confirmed. We order bleedings, emetics, purging draughts, yea whole claſſes of the materia medica, not with any ſatisfactory reaſon adapted to the animal œconomy, but becauſe authors of a great name have recommended them; and we pervert faith (certainly a great Chriſtian virtue) by applying it to a ſcience where it ceaſes to be one.

I ſhall at this time endeavour to ſhew, in a few lines, that we miſunderſtand one of the moſt common appearances, and that it leads us, in conſequence, to a wrong mode of practice, I mean *a foul tongue*. It is adopted nearly as an axiom in the ſchools, that a loaded tongue indicates a foul ſtomach, and it is therefore no longer matter of enquiry whether this concluſion is true or not. Notwithſtanding the prevalence of the opinion, I hope to ſhew its error.

Suppoſing the ſtomach overloaden even with its own corrupted juices, or with matters taken in by the mouth, could it happen, againſt the laws of gravity, that ſome particles of them could aſcend, to depoſit themſelves upon the tongue, ſuppoſe to ſhew us, that the ſtomach contained corrupted or more matter only than it ſhould do? Certainly not.

Suppoſing the laws of gravity had ſuffered an alteration in favour of phyſicians and apothecaries, to give them occaſion to adminiſter their tartariſed antimony, how ſhould theſe matters eſcape the ſtomach, becauſe it is well known that the mouth of the ſtomach is always cloſely ſhut, and that for this reaſon we cannot even diſcharge air without a great effort; though air ſhould find its way eaſier than the black viſcous ſtuff we often remark upon the tongue.

We find our tongue clean after dinner, and unclean when we we awake, and when our ſtomach is quite empty, and in ſo ſound a ſtate that we feel hungry, and order our breakfaſts. The ſtate of a ſtomach overcharged with victuals, or containing corrupted juices, cannot laſt ſo long as we often remark a foul tongue to continue. The acrimony of the matter would increaſe, irritate the ſtomach, and cauſe vomiting, or find its way downwards.

Another error generally entertained by the public, and too often by practitioners, confirms the one I am ſpeaking of. As ſoon as they ſee an unclean tongue, an emetic is pronounced unqueſtionably neceſſary. They adminiſter it, and with the ſecond or third vomiting always comes forth ſome bile, and ought to come, of whatever conſtitution the patient may be, provided his biliary duct is not obſtructed by calculas. Now they conclude, often out of ignorance, often out of quackery, the beneficial effects the emetic has produced! From what violent diſeaſes it has ſaved the patient!

And to what practitioner has it not happened to obſerve ſpontaneous vomiting very frequent, eſpecially in this ſeaſon of the year, ſoon after the great heat, of green bile, and ſo corrupted that it corrodes the fauces, and feels acrid in the mouth, while the tongue is as clean as poſſibly it can be. I have had an opportunity of obſerving this, within theſe few days, in a boy of eleven years old, ſon of a French emigrant, who had a fever, accompanied with head-ach, loſs of appetite, and pains, with great uneaſineſs, in the region of the ſtomach, &c. *but a red, clean, humid tongue.* I preſcribed for him an emetic, and he vomited immediately *green*, acrid bile, after which he recovered. His tongue was, before, during, and after the indiſpoſition, of a true bilious kind, always in the ſame ſtate.

It ſeems, upon the whole, that we accuſe the bowels too often as a cauſe of many indiſpoſitions, becauſe we do not endeavour to find any other.

The paſſage being always ſo largely open, and few cauſes exiſt which may obſtruct it, it ſeems not likely that crudities could be ſo often accumulated as we ſuſpect them; and I think, that the unſatisfactory conſequences of our too frequent purging draughts would inform us of the error, if the majority of practitioners believed leſs in authorities, and accuſtomed themſelves more to make obſervations.

Thus, I believe that I have proved, a priori, that the ſymptom of an unclean or furred tongue, never *can* proceed from a foul ſtomach only; and it has the more weight, as it is confirmed by diſſection.

Having lived in Italy for ſeveral years, I have often taken the advantage of the facility of diſſecting bodies in that country; and putrid fever being one of the moſt common cauſes of death in Italy, eſpecially during the five hot months, I have laid open the ſtomach and bowels of a great number who died with a furred, even with a black tongue, but never found any crudities in them.

I commonly knew the remedies they had taken during their illneſs, and it ſeldom is the practice in Italy to give an emetic or purging draught in that diſeaſe; but in both caſes I found the inteſtinal canal clear.

Theſe reaſons ſhew clearly that it is impoſſible a foul tongue ſhould ſhew the ſtate of the ſtomach.

There muſt therefore be another ſource for this ſymptom, which is ſo frequently remarked, and this, in my opinion, is twofold, namely:

It is either,

1. A precipitation from the ſaliva; or,

2. An effect of increaſed evaporation.

If it proceeds from the decompoſition of the ſaliva, then our food becomes bitter to the palate, for the ſenſe of taſte does not perceive it till diſſolved; or they have ſome particular taſte, which in a natural ſtate of that fluid they ſhould not have: In ſhort, the ſpittle comes then nearer its corrupted ſtate than it ſhould do in a ſound perſon.

If it proceeds from a precipitation of the breath, then this will be in general offenſive, from being loaden with heterogenous matter, which, by its paſſage through the mouth, is in part precipitated upon the tongue, gums, and teeth. In this caſe it is a ſign of the ſtate of the blood, and of the functions of the lungs.

It is certainly very neceſſary for the progreſs of the healing art, to doubt ſometimes what the ſchools of phyſic, during centuries, have acknowledged as facts, to abandon nonſenſe, and to ſeek the truth.*

*London, Sept.* 1800. W. DOMEIER.

---

* The ſentiments and reaſoning of this Correſpondent, about ſixty years ago, would have procured him fame; at preſent the Humorial Pathology of Boerhaave is become obſolete in this country. The appearance of the tongue, however, is a circumſtance of ſo much importance, that we wiſhed to call the attention of our Correſpondents to the ſubject, and have therefore admitted the paper.——EDIT.

